# Road Surface Monitoring Using Smartphone Sensors: A Review

**DOI:** 10.3390/s18113845

**Published:** 2018-11-09

**Authors:** Shahram Sattar, Songnian Li, Michael Chapman

**Affiliations:** Department of Civil Engineering, Ryerson University, Toronto, ON M5B 2K3, Canada; shahram.sattar@ryerson.ca (S.S.); mchapman@ryerson.ca (M.C.)

**Keywords:** smartphone sensors, road surface anomaly, crowdsourcing

## Abstract

Road surface monitoring is a key factor to providing smooth and safe road infrastructure to road users. The key to road surface condition monitoring is to detect road surface anomalies, such as potholes, cracks, and bumps, which affect driving comfort and on-road safety. Road surface anomaly detection is a widely studied problem. Recently, smartphone-based sensing has become increasingly popular with the increased amount of available embedded smartphone sensors. Using smartphones to detect road surface anomalies could change the way government agencies monitor and plan for road maintenance. However, current smartphone sensors operate at a low frequency, and undersampled sensor signals cause low detection accuracy. In this study, current approaches for using smartphones for road surface anomaly detection are reviewed and compared. In addition, further opportunities for research using smartphones in road surface anomaly detection are highlighted.

## 1. Introduction

Recently, the monitoring of road surface conditions has become considerably important. Well-maintained road surfaces increase road user safety and comfort levels. Therefore, it is essential to monitor road conditions continuously to enhance the transportation system in terms of driving safety and comfort. For instance, in Canada, authorities responsible for road surface maintenance have to deal with complaints concerning the poor surface conditions of roadways, particularly during the winter months. One of the main indicators used to determine road surface conditions is the density of road surface anomalies [1]. Municipalities typically rely on statistical data derived from collected road surface information, visual field inspections, or vehicles outfitted with special instruments which measure and monitor road surface conditions. For example, ARAN (Automated Road Analyzer), which is widely used for road monitoring in Canada, and ROMDAS (Road Measurement Data Acquisition System), which is used to monitor road surfaces in New Zealand, both use lasers combined with ultrasonic and video sensors for high-level road quality assessment [1]. However, these methods are labor-intensive, costly, and often suffer from insufficient data coverage to generate a complete picture of road surface conditions in large cities, such as Toronto, Canada. For the above reasons, road maintenance authorities are looking for a low-cost, higher-efficiency detection method. They also desire a centralized information system able to monitor the road status in real time. Traditionally, there have been three main approaches for road surface monitoring: 3D reconstruction, vibration, and vision-based [2].

A 3D reconstruction approach relies on 3D laser scanning to create accurate surface models. These models are then compared to a base model to detect road surface anomalies. In this approach, a 3D laser scanner uses reflected laser pulses, which create accurate 3D digital models of existing objects, such as road surface anomalies. Subsequently, the distress features (road surface anomalies) are extracted from the created point clouds (i.e., a collection of points that represent a 3D shape of road surface distress). This approach was widely investigated by Kelvin [3], Vijay and Arya [4], Salari et al. [5], Moazzam et al. [6], Hou et al. [7], Kim and Ryu [8], Wang et al. [9], and Yan et al. [10]. However, the aforementioned approaches require high-cost laser scanners [8] and are very costly when monitoring large-scale road networks.

A vision-based approach relies on image processing analysis, such as texture extraction and comparison using captured photographs depicting pavement distress features. The principle of this approach primarily utilizes geotagged images captured by a camera/video system mounted facing downward toward the road surface on a moving vehicle. Any suspicious road surface distress features, including potholes and cracks, can be automatically detected from the collected geotagged video images [11] by applying, for example, a Canny edge detection process [12]. Vision-based approaches were extensively evaluated by Koch et al. [13], Jog et al. [14], Huidrom et al. [15], Lokeshwor et al. [16], and Yan and Yuan [11]. Even though these approaches are cost-effective compared to 3D reconstruction approaches, they depend on certain environmental conditions, such as lighting and shadow influence, etc.

With a vibration-based approach, road surface anomalies are detected from the rate of moving vehicles’ vibrations captured by motion sensors (e.g., accelerometers or gyroscopes). Theoretically, a vehicle, when passing over any road surface anomaly, such as a pothole, crack, manhole, or expansion joint, will vibrate more than when passing over smooth road surfaces.

Over the last few years, smartphone-based sensing has become an important supplemental technique for detecting road surface anomalies in order to monitor the surface of the road segments or the surface of bicycle and pedestrian lanes [17]. Participatory sensing is anticipated to be an emerging area in which smartphone-based measurements seem to be particularly attractive, as they are not only widespread but also equipped with several sensing capabilities [18]. Measuring and analyzing motion sensors’ signal data from various types of moving smartphones may diverge depending on many factors, including the sensor’s characteristics and quality, smartphone device’s position, vehicle’s suspension system, and speed [19].

This paper aims to provide a comprehensive review of the existing studies that investigate how smartphone sensors may help detect road surface anomalies by synthesizing the problems, major issues, and challenges in current development, as well as identifying the existing research voids for further research studies. Therefore, the inclusion criteria for the articles in this paper were the evaluation of existing approaches using smartphone sensor data for road surface anomaly detection. In addition, articles that have no focus on the application of smartphone sensors or those using handheld computer devices other than smartphones, such as PDAs (personal digital assistants), or those using low-end accelerometer kits were excluded from this review. It is important to note that there are other studies that also address the application of monitoring road and traffic conditions using smartphones. However, only the parts of these studies that relate to the scope of this paper are considered and reviewed.

This paper is organized as follows. Section 2 compares related work according to the overall workflow of detecting road surface anomalies from smartphone sensors, followed by a review of parameters affecting the performance of the detection and integration approaches. Section 3 presents a brief discussion of the reviewed articles and their limitations as a comprehensive approach. Conclusions and the challenges of detecting road surface anomalies from smartphone sensors, as well as potential future research areas, are given in Section 4.

## 2. Related Work

Measuring the signals from smartphone sensors is challenging due to dissimilar sensor properties between various models of smartphones, as well as differences in vehicle size, weight, length, and suspension systems. There is also a difference in a vehicle’s vertical motion induced by road surface anomalies—the length, depth, and shape of the potholes on the road surface and the road curvature’s effect on the vertical movement rate of different vehicles. In fact, different vehicles passing over a specific pothole would not generate an identical signal pattern [20]. In addition, vehicle velocities affect the vertical movement rate, which then leads to different patterns of the vibration response to any road surface anomaly.

**Figure 1:**
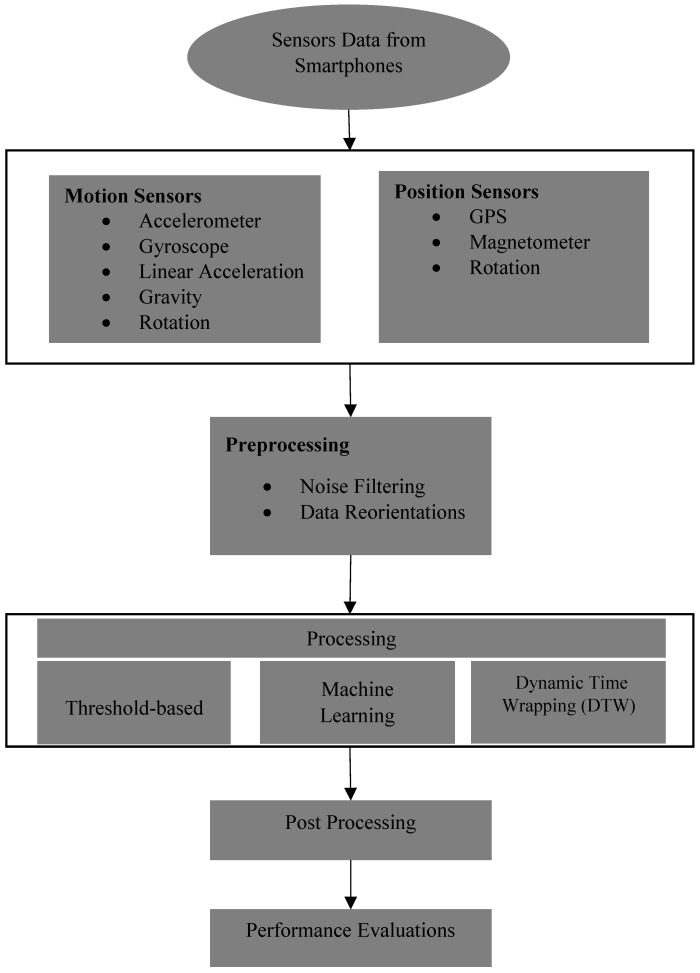
Road surface anomaly detection process.

Road surface anomaly detection approaches consist mainly of five steps: (1) sensing (data collection), (2) preprocessing, (3) processing for feature extraction, (4) post-processing, and (5) performance evaluations. Figure 1 illustrates the overall process for road surface anomaly detection using smartphone sensors.

There are different kinds of sensor data that can be obtained from smartphone sensors. Motion sensor types include: accelerometer, gyroscope, linear accelerometer, and rotation. Position sensor types include: GPS, manometer, and rotation. The next step is the preprocessing of sensor data, with the aim of filtering noises that contaminated the sensor data. The other goal of preprocessing sensor data is to reorient them from the device coordinate system to another geographic coordinate system. The preprocessed sensor data are then analyzed to discover and extract desired information based on predefined rules (feature extraction). After that, the processed sensor data should be transferred to the central server for data post-processing, including integration with other data from different sources (concept of crowdsourcing). Finally, the performance of the proposed process should be evaluated to determine its functionality and reliability.

**Sensor Data Collection**

The smartphone sensor framework has open access to many types of built-in sensors. Some of these sensors are hardware-based (physical) and some are software-based (virtual). Hardware-based sensors are the physical, built-in sensors, such as accelerometers, gyroscopes, magnetometers, light, temperature, etc. Physical sensors measure motion, orientation, and environmental conditions, such as acceleration force, physical position of device, illumination, etc. In contrast, software-based sensors use data from one or more of the hardware-based sensors and virtually calculate real-time values based on the desired outcome, such as linear acceleration, rotation, gravity, etc. In general, smartphone sensors can be categorized into three different types: motion sensors, position sensors, and environmental sensors.

**Figure 2:**
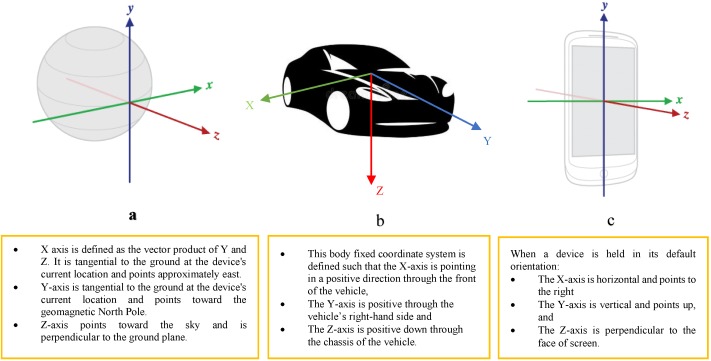
(**a**) Local-level coordinate system. (**b**) Body-frame coordinate system. (**c**) Device coordinate system.

Motion sensors are suitable for monitoring a device’s movement and vibration, tilt, shake, rotation, or swing. The movements can directly reflect user interaction, as typically happens in game applications (i.e., a user steering a car or a controlling a ball in a game). However, they can also reflect where the device is sitting (i.e., moving with the occupant while they drive their car). With direct user interaction, device movement is monitored relative to the device’s coordinate system or a defined local application frame. With physical environmental conditions, the device movement is monitored relative to the local-level coordinate system (Figure 2).

Position sensors are suitable for specifying a device’s physical position in the local-level coordinate system. In fact, the geomagnetic field sensor, in combination with accelerometer sensor data, can determine a device’s position relative to the local-level coordinate system. Environmental sensors measure the environmental properties of the surrounding area, such as temperature, humidity, ambient pressure, and illuminance. This type of sensor has a very limited contribution to road surface anomaly detection, since there does not seem to be any direct relationship/impact between these factors and the formation of road surface anomalies.

For each smartphone-based application, various combinations of sensors (physical or virtual) may be used depending on the desired application criteria. To develop an application for road surface anomaly detection, motion sensor data can be tracked to detect any possible shake or tilt caused by road surface anomalies in a moving vehicle. Previous studies investigating road surface anomaly detection using smartphone sensors have widely employed motions sensors (accelerometers and gyroscopes). Accelerometer sensors measure acceleration force, including gravity force, applied to a device on all three physical axes (m/s^2^). Gyroscope sensors measure a device’s rate of rotation around each of the three physical axes (rad/s). Previous studies have frequently used accelerometer sensor data to detect anomalies because road surface anomalies have more influence on and are mainly detectable from the acceleration force applied to the vehicles, rather than the rotation rate caused by vehicles’ vibration. Only a few studies, including Yagi [21] and Douangphachanh and Oneyama [22], have investigated gyroscope sensor data, particularly frequency domain combined with accelerometer sensor data, which increases the accuracy of detection (as complementary sensor data).

To determine the current location information of smartphone or mobile devices, including latitude, longitude, bearing of moving direction, and velocity of movement, the location API (application program interface) provides the best available location information of the devices based on the currently available location providers, such as GPS (Global Positioning System) and/or Wi-Fi (Global Positioning System). In addition, the API provides the accuracy for each provided location information data.

Table 1 summarizes and compares a list of sensors commonly found in smartphones for the application of road surface anomalies [23]. These sensors are used either directly or indirectly for road surface anomaly detection.

**Pre-Processing**

The data preprocessing step involves transforming raw data derived from smartphone sensors into a clean and organized data set prior to analysis [24]. One of the goals of preprocessing is to smooth the raw sensor data and filter the noise. There are three major types of smoothing and filtering approaches: moving-average filtering, low-/high-pass filtering, and band-pass filtering. Moving-average filtering is the most common filter in digital signal processing, mainly because it is the easiest to understand and implement since there is no need to have any prior information regarding the sensor data [25]. Despite its simplicity, this kind of filter is ideal for some common tasks, such as reducing random noise while retaining major information content. Low-/high-pass filters remove some undesired parts of signals based on a predetermined cut of frequencies. A band-pass filter passes portions of the signals within a certain range of frequencies and removes the other parts of signals that are outside of that range.

Preprocessing may also aim to reorient sensor data values from a device coordinate system (Figure 2c) to the local-level coordinate system (Figure 2a). An example would be a body-frame (i.e., moving platform) coordinate system (Figure 2b) [26]. This process can be accomplished by completing a rotation (using Euler angles) around each of the three axes. The reorientation process reduces issues related to smartphone placement.

**Processing**

The processing step analyzes the preprocessed sensor values to detect road surface anomalies. The signal pattern is tracked to detect any abnormal changes in sensor values using three main approaches:

1. The threshold-based approach uses simple predefined threshold values based on experiments to detect road surface anomalies from sensor data.
2. The machine learning approach uses more advanced techniques to detect road surface anomalies. Studies Bhoraskar et al. [27] have investigated unsupervised approaches, such as k-means clustering, in which a predetermined number of clusters are identified, and data are classified to the same number of clusters. Some other studies, including Perttunen et al. [28] and Jain et al. [29], have explored supervised approaches, such as support vector machine (SVM) clustering. In the case of supervised approaches, some training data sets should be collected to train the algorithm. The test data are then classified based on the trained data set.
3. Another approach that was recently investigated is dynamic time warping (DTW). This approach is predominantly employed in speech recognition studies. DTW compares incoming signal data with predefined templates and measures the similarity between the data sets.

Regarding smartphone applications, the preprocessing and processing steps can be accomplished using two different modes: online and offline. In an offline mode, sensor data are collected, and the preprocessing and processing steps are applied locally on computers. In fact, any application developed for sensor data collection is able to collect and store sensor data while a car is passing over potholes or bumps. Next, the sensor data are extracted for further processing. In an online mode, data collection, preprocessing, and processing are performed simultaneously as the car is passing over potholes or bumps. Ideally, a specific smartphone application should be designed and developed to collect, preprocess, and process the sensor data in an online mode.

**Post-Processing**

Post-processing includes crowdsourcing and integrating data from multiple sources (users’ collaborations, geographic datasets) to increase the accuracy of detection and scalability. Detection results from various users can be integrated (data fusion), enabling more reliable and precise detection. Due to the dynamic behavior of road surface anomalies, integrating the detection results from various users at different times can help evaluate a road surface anomaly’s condition more precisely than in the spatiotemporal domain. Moreover, other geographic data, such as road networks, manhole, and catchment basin location data, can be integrated through filtration or data analysis to increase the accuracy of the results. As an example, manholes and road joints behave similarly to road surface anomalies in sensor data. Therefore, if the geolocation of man-made anomalies is integrated, sensor-detected road surface anomalies (i.e., the manholes or joints in this case) are subsequently able to be filtered.

The central server stores incoming data while also processing parallel incoming data from multiple sources. The completed central processed data can be presented as a geospatial information system (GIS) web-based map to the general users or authorities dealing with road surface maintenance.

### 2.1. Sensor Data Collection

A major step in developing a viable approach that can detect road surface anomalies is the collection of sample sensor data from smartphones’ sensors. As discussed earlier, motion sensors, such as accelerometers and gyroscopes, have been widely used in the collection and processing of data for road surface anomaly detection. Data collection from previous studies (refer to Table 2) differ in terms of the types of sensors being employed, sampling rates, variety of vehicle, and devices considered for the data collection. These indicated factors are the most critical parameters that impact the performance of a smartphone’s ability to detect road surface anomalies. Table 2 summarizes the sensor data collection properties reviewed in the selected studies.

According to Table 2, accelerometer sensors have been widely investigated as a means to develop an approach to detect road surface anomalies. In most previous studies, accelerometer sensor data were investigated in the time domain for detecting road surface anomalies. However, gyroscope sensor data were transformed to the frequency domain for feature extraction (road surface anomaly detection). Moreover, most of the previous studies only employed accelerometer sensors to detect road surface anomalies. However, Yagi [21], Douangphachanh and Oneyama [22], and Mohamed et al. [30] attempted to combine gyroscope and accelerometer sensor data to increase detection accuracy using a data fusion technique.

Data sampling rates play a significant role in the processing of any detected event. Choosing an appropriate sampling rate is a design decision affected by multiple factors, such as: available resources, required accuracy, and the type of data being used for event recognition [31]. As an example, if only frequency domain features are used for monitoring road anomalies, the sampling rate should be high enough to capture all relevant frequencies. According to Douangphachanh and Oneyama [32], the road anomalies most likely have the frequency range of 40–50 Hz are captured in accelerometer data.

A higher sampling rate increases the chances of capturing and detecting road surface distress features. However, it also increases the battery usage of a smartphone, as well as the required capacity to store and process data. Finding proper sampling rates is related to the speed of movement, as well as the mechanical properties of a vehicle. Sinharay et al. [19] investigated the use of a low sampling rate to develop their approach to road surface anomaly detection.

Various models of vehicles and smartphone devices are factors considered by previous studies when collecting data. According to Table 2, Douangphachanh and Oneyama [22] and Jain et al. [29] studied both different vehicles (e.g., sedan, SUV, trucks) and smartphones (i.e., different manufactures) to ensure their approaches functioned equally in different circumstances.

### 2.2. Sensor Data Preprocessing

The preprocessing of sensor data values is important for two major reasons: filtering noise that distorts parts of the signal; data cleaning and sensor data reorientation. Not all the reviewed studies preprocessed the sensor data. Some reviewed studies only conducted noise filtering and data smoothing approaches as their preprocessing step while some other studies only conducted signal data transformation before processing them. Sebestyen et al. [33] and Seraj et al. [34] utilized two different filters: one for eliminating noise, and one for amplifying acceleration variation caused by road anomalies. Douangphachanh and Oneyama [22] used a high-pass filter to detect low-frequency information, such as changing speed and vehicle maneuvering and turning, which have lower frequencies than road surface anomalies from sensor data. Mohamed et al. [30] applied the second order high-pass butterworth filter proposed by Butterworth [35]. Harikrishnan and Gopi [36] collected data segmented into groups of n-samples. Then, a filtering process was conducted to preserve data samples induced by potholes or speed bumps, as well as to minimize the parts of sensor data corresponding to normal roads. To smooth the signal data, Singh et al. (2017) applied a simple moving-average and band-pass filter to smooth accelerometer sensor data values before processing them. The approaches proposed by Mohan et al. [37], Bhoraskar et al. [27], Vittorio et al. [38], Sebestyen et al. [33], Wang et al. [9], and Singh et al. [39] entailed the application of Euler angles (rotation angles) calculated from accelerometer sensor data to transform the signal data values from device coordinate systems to the local-level coordinate system orientation using a coordinate transformation technique. Silva et al. [40] applied a data cleaning process by eliminating all null sensor data values and inconsistent timestamp data values before processing them.

### 2.3. Sensor Data Processing

Processing sensor values for the application of road surface anomaly detection has three main approaches: threshold-based, machine learning, and DTW. Table 3 summarizes the approaches used by previous studies when processing sensor data and detecting abnormal changes in signal data. Data processing in this application can be reviewed in terms of the feature extraction approach (e.g., threshold-based, machine learning, and DTW), ability to classify road surface anomalies (anomaly classification capability), smartphone orientation dependency in detection, and speed dependency in detection.

#### 2.3.1. Feature Extraction Approach

**Threshold-Based Approach**

In order to detect road surface anomalies using threshold-based approaches, sensors’ changing data value patterns or statistical values (e.g., standard deviations) taken from sensor data values were analyzed. The amplitude of the accelerometer signal was monitored and the anomaly’s patterns in the signal were identified (an anomaly’s patterns in digital signals occur when the power of the signal exceeds a specific value). Threshold-based approaches were reviewed from three different perspectives: length of interval for a window function, fixed vs. flexible threshold determination, and amplitude of signal vs. other properties of signal amplitude (e.g., mean and standard deviation).

Determining the interval length for a window function in spectral analysis is challenging, as it is related to various factors, such as the speed of vehicles and the distance from the front to rear wheels. A window function considers predefined intervals of the signal data for analysis and feature extraction as opposed to looking at signal data individually. Table 3 summarizes the length of interval for a window function for each of the studies that explored window functions. Defining proper threshold values in a statistical approach is an intensive process since road anomaly data values are affected by variable conditions. The suspension system of a car, sensor properties of smartphones, and smartphone placement all affect how smartphones sense a single anomaly. Studies conducted by Mohan et al. [37], Mednis et al. [41], Sinharay et al. [19], and Yi et al. [43] determined fixed-threshold values from experiments studying road surface anomaly detection. However, studies conducted by Sebestyen et al. [33], Wang et al. [9], and Harikrishnan and Gopi [36] utilized dynamic threshold values to overcome unsteady signal patterns caused by various sensor and mechanical properties. Dynamically assigned threshold values are desirable when creating methods for detecting road surface anomalies, as they can be adapted to different circumstances.

Mohan et al. [37], Mednis et al. [41], Sebestyen et al. [33], Wang et al. [9], and Harikrishnan and Gopi [36] determined thresholds based on the amplitude of the signal. However, other studies, such as Yagi [21], Nomura and Shiraishi [42], Vittorio et al. [38], and Yi et al. [43] determined thresholds based on the statistical values (such as the standard deviation) derived from signal values. Mednis et al. [41] confirmed that the standard deviation is the most important parameter for detecting road surface anomalies from accelerometer sensor data.

**Machine Learning Approach**

There are two prevalent approaches using machine learning techniques: supervised learning and unsupervised learning [44]. The reviewed studies that involved machine learning techniques can also be categorized based on these two approaches. Bhoraskar et al. [27] used k-means, an unsupervised method, to classify sensor data on smooth and bumpy roads, as well as to train the SVM algorithm. In this approach, the classes (bumpy or smooth) were manually labeled. Moreover, Mohamed et al. [30] utilized the proposed threshold-based approach to label classes (smooth or speed bump) and train an SVM algorithm with three different kernel functions to distinguish between bumps and smooth road. Perttunen et al. [28], Jain et al. [29], Seraj et al. [34], and Mohamed et al. [30] employed SVM to classify sensor data. Captured videos, images, sounds, or field inspection were utilized to collect the ground truth and label the anomalies for classification purposes. Silva et al. [40] investigated the performance of various supervised machine learning approaches, such as gradient boosting (GB), decision tree (DT), multilayer perceptron classifier (MPL), Gaussian Naive Bayes (GNB), and linear SVC to classify road surface distresses into different classes, including short bump, long bump, unleveled manholes, and others. Although these methods successfully classified the sensor data, a sample of labeled data was required to train the supervised algorithm first, which is impractical for real-time or near real-time applications.

**Dynamic Time Warping Approach (DTW)**

In time series signal processing, the DTW approach measures the similarity between any two patterns of signals and extracts features from signal data [45]. For example, Singh et al. [39] proposed a DTW-based approach to detect road surface anomalies from accelerometer sensor data. In this approach, time series values captured accelerometer sensor data for every pothole and bump, and the data were then stored in a central server as templates. Next, incoming sensor data were compared with the stored templates to detect similarities. The accuracy of this approach was greatly correlated to the quality of the reference template. Therefore, this approach was both computationally intensive and unreliable, as it required reference templates for each different condition (i.e., various vehicles, road conditions, speed of driving).

#### 2.3.2. Differentiating Various Forms of Road Surface Anomalies

Anomalies existing on road surfaces can be categorized into two major forms:

1. Actual road surface anomalies, including potholes and cracks;
2. Man-made road surface anomalies, including manholes, road joints, catchment basins, and speed bumps.

A comprehensive pothole detection approach should be able to differentiate actual road surface anomalies (such as potholes and cracks) successfully from a variety of man-made anomalies (such as manholes and speed bumps). However, this is challenging as they both generate similar signal patterns, especially in the case of manholes and catchment basins.

In an approach proposed by Sebestyen et al. [33], potholes can be distinguished from man-made speed bumps. If a car runs over a pothole, the car first drops and then climbs back up. Conversely, if a car runs over a man-made bump, the car first climbs and then drops. Therefore, by setting these rules within the signal pattern, these anomalies were detected and separated. Sinharay et al. [19] suggested that the standard deviation of values calculated from sensor data can be used to distinguish potholes from bumps. Seraj et al. [34] classified detected road surface anomalies to three different classes: severe anomalies, mild anomalies, and spans. Harikrishnan and Gopi [36] used an X-Z filter proposed by Eriksson et al. [46] to differentiate between potholes and speed bumps. Eriksson et al. [46] claimed that potholes are mainly caused by an impact on one side of the vehicle, resulting in a relatively large variation on the x-direction of the accelerometer sensor data. However, speed bumps cause an impact on both sides of a vehicle, leading to small variations on the x-direction of accelerometer sensor data values. Such a mechanism can then be used to distinguish between potholes and speed bumps.

#### 2.3.3. Smartphone Orientation Dependency

Road anomaly detection results are sensitive to the sensors’ orientation. Most of the reviewed studies, such as Yagi [21], Mednis et al. [41], Perttunen et al. [28], and Sinharay et al. [19], assumed fixed and predetermined positions for analyzing smartphone sensor data. They required users to place their mobile device at a specific orientation and restricted them from using their mobile devices freely. As such, the smartphones had a lack of orientation independence. In order to find a practical road surface anomaly detection solution, smartphones should be freely placed. To develop an approach independent from smartphone orientation, two methods have been investigated:

- **Signal transformation:** In this method, the sensors’ values are transferred from the device coordinate system to another geometric coordinate system (e.g., local-level coordinate system or body-frame coordinate system).
- **Orientation-independent features:** In this method, the magnitude of the sensor data values on all three axes is considered instead of considering their individual values on three separate axes.

The methods proposed by Mohan et al. [37], Bhoraskar et al. [27], Vittorio et al. [38], Sebestyen et al. [33], Wang et al. [9], and Singh et al. [39] applied a signal transformation method, which uses the Euler angles computed from accelerometer sensor data for coordinate transformations. Conversely, the approaches proposed by Jain et al. [29], Sinharay et al. [19], and Yi et al. [43] utilized orientation-independent features of acceleration data (i.e., vector sum, mean, standard deviation) to become independent from smartphone orientation.

#### 2.3.4. Speed Dependency

Another factor that influences road anomaly detection using smartphone sensors is the speed of the vehicle. Douangphachanh and Oneyama [32] demonstrated that average speed plays an important role in road roughness estimation. When a car passes over a specific road anomaly, such as a pothole, at different speeds, the amplitude of the collected acceleration signal reacts in a different manner, which should be modeled [47]. Fox et al. [20] investigated the effect of velocity as a component for detecting road surface anomalies from an onboard accelerometer sensor. Their investigations revealed that at high speeds, discriminating between normal roads and pothole regions was difficult. Sebestyen et al. [33] collected sensor data at three different speeds: 15, 30, and 60 km/h. All values from the different speeds were normalized to a value of 30 km/h to develop and verify the proposed approach. Yi et al. [43] examined the effect of vehicle velocity discussed in their approach by creating a lookup table, as well as categorizing the speed into different ranges. Then, each event was indexed according to the ratio of standard deviation, as well as the standard deviation of stable periods of the speed interval during which the event had been detected. Seraj et al. [34] used speed data logged from the GPS sensor to demodulate accelerometer sensor data and amplify part of the signal caused by anomalies by considering the speed of the vehicles. Sinharay et al. [19] normalized the feature values based on the speed of the vehicle. Speed was categorized in three ways: lower than 2 km/h, between 2 and 30 km/h, and more than 30 km/h. In addition, Perttunen et al. [28] adopted an approach by Tanaka et al. [48] that removed the effects of speed on signal data. Mednis et al. [41] used different algorithms for different speeds. For instance, for a speed of less than 25 km/h, the z-sus algorithm was implemented. For the speed of more than 25 km/h, the z-peak algorithm was implemented. Mohan et al. [37] used the z-sus method for speeds of less than 25 km/h and z-peak for speeds of more than 25 km/h. To minimize the false positive detection rate, Harikrishnan and Gopi [36] proposed specifying a velocity-dependent variable. This variable was adjusted to the threshold value based on the current velocity of the vehicle. Different studies have used various methods to deal with the effects of vehicle speed on the performance of their approaches to road anomaly detection. However, none of them provided a technique robust enough to account for the effect of a vehicle’s velocity.

### 2.4. Post-Processing of Detected Events (Road Surface Anomalies)

In this section, studies that investigated data integration and approaches used to process detected road surface anomalies from various users (i.e., data fusions) are reviewed and discussed. Chen et al. [49] and Fox et al. [20] transferred identified potholes’ information for each selected region in their study to the cloud for further analysis in their proposed approach. Then, a voting algorithm was applied to the study area for final decision making. In fact, the voting algorithm counts the number of reports made for each phenomenon from different sources. If the number of reported anomalies from various sources, for each predefined slice of the road, is more than a predefined threshold, those anomalies are considered true detection. Otherwise, they are rejected and assumed false detection. Fox et al. [20] considered a sliding window of 10 m for evaluating the number of reports from smartphones on-board vehicles in order to minimize the false positive rate of detection.

Unfortunately, for both studies, this simple voting algorithm ignores the fact that sources have different degrees of trustworthiness [50]. In addition, this binary-based algorithm does not consider the temporal and probabilistic nature of the anomalies. Results conducted by Fox et al. [20] indicated that at least 10 vehicles operating at a speed of 50 km/h were required for data collection in order to reach an accuracy of 90%. Furthermore, Chen et al. [49] claimed 90% accuracy with nearly zero false positive alarms for their crowdsourcing-based road surface monitoring (CRSM) system.

Yi et al. [43] adopted a grid-based clustering algorithm called DENCLUE (DENsity CLUstering) to filter out false detections using the reporting frequency of events in a 5-m grid zone. Neighboring grids were grouped together if the frequency of the reported anomalies for each neighbor grid was more than three. Otherwise, the grid was assumed to be noisy and was removed. The drawback of this strategy is that some anomalies close to each other were treated as a single anomaly. In addition, threshold-based approaches for classifications are similar to the voting algorithm, which suffers as a result of considering the temporal and probabilistic nature of any road surface anomaly detecting by smartphones. Moreover, there is always a trade-off between reducing the false detection rate and missing the detection of anomalies at the same time. In this study, only the position accuracy of detected road surface anomalies was investigated, and the overall accuracy of detection was not studied.

Alessandroni et al. [51] proposed the “SmartRoadSense” system. Roughness information was collected in a central server. Then, the average of all roughness values within the predefined buffers of the detected location was considered to be the roughness value for that region. Sebestyen et al. [33] proposed a method in which an average was taken to combine incoming information from multiple users. In this method, a weighted sum between the available evaluations was computed, as well as integrated multiple surveys from various incidents. This method, similar to the voting algorithm, suffers from the ignorance of the temporal aspect of road surface anomalies and the inherent uncertainty of incoming data. In addition, defining the proper buffer distance is challenging because it significantly affects the detection rate.

### 2.5. Performance Evaluations

To evaluate the performance of road surface anomaly detection approaches, performance metrics are required, including accuracy ratio, precision, false positives ratio, and false negatives ratio. The choice of a specific performance metric or a combination of different performance metrics depends on the type of application needed, as well as its performance requirements. Table 4 summarizes overall performance evaluations for each approach based on the provided performance metrics. In addition to the overall accuracy of the analysis, some studies investigated performance evaluations with different smartphone placements.

**Smartphone Placement**

One of the challenges in road surface anomaly detection is that smartphone sensors are sensitive to the placement of the device. In most studies, the location of mobile phones was considered to be fixed to a mount on the windshield or attached to the dashboard. However, few studies have investigated the performance of their approaches with smartphones in different locations in the vehicle, such as in the driver’s pocket or in the console near the gearbox. Different drivers have different habits, and the ideal approach should consider any circumstance that could result in a change in the location of a smartphone and its impact on the loss of recognition performance. Table 5 indicates that only three studies investigated the performance of their respective approaches with different smartphone placements in moving vehicles, namely, [29,32,43]. Douangphachanh and Oneyama [32] confirmed that smartphones located in a driver’s pocket or in the console caused lower detection rates.

## 3. Discussion

In this review, the existing research investigating approaches for road surface anomaly detection using smartphone sensors is reviewed and compared. The existing approaches are compared using five primary criteria: sensor data collection, preprocessing, processing, post-processing, and performance evaluations.

Data collection is one of the most important considerations when developing any approach to road surface anomaly detection with smartphone sensors. It is essential that the detection process considers all relevant aspects, including smartphone sensor properties, smartphone mounting location, vehicle suspension system, driving behavior, and speed. Therefore, to guarantee that an approach is compatible with different conditions, sensor data should be collected in various situations: various models of cars, smartphones, and speeds. However, due to the limitation of resources, only certain vehicles and smartphone devices have been selected and used for data collection in the reviewed research studies.

Preprocessing is also an important task for any application using sensor data to extract features such as road surface anomalies. Preprocessing has two major objectives:

1. To smooth the sensor data, to amplify the parts of the sensor data caused by the event (road surface anomaly), and to attenuate or remove parts of sensor data caused by noise or undesired input.
2. To reorient the sensor data from a device coordinate system to the body-frame (vehicle coordinate system) or local-level coordinate system.

In fact, the preprocessing step can increase the accuracy of detection and decrease the false detection rate. In most reviewed studies, it was assumed that the general location of a smartphone was in a rigid holder. It was also assumed to be fixed, both position- and rotation-wise, with respect to the body-frame (vehicle) coordinate system. Unfortunately, when the smartphone is positioned in its holder or placed on the dashboard, there is no guarantee that the direction of the sensor data measurements will align with the vehicle’s body-frame coordinate system. The relative direction between the device’s coordinate system and the body frame’s coordinate system is therefore considered unknown [52]. In fact, the desired approach should give freedom to users concerning smartphone placement. Several studies have considered sensor data reorientation using accelerometer sensor data to approximate rotation angles (employing Euler angles). However, the calculated rotation angles from accelerometer sensor data are both biased and contaminated by variant noise caused by thermal and mechanical fluctuations inside the sensor. Most modern smartphones using microelectromechanical sensors (MEMS), such as inertial measurement units (IMU), which contain a three-axis gyroscope for measuring angular velocities around three axes (i.e., pitch, roll, and heading), a three-axis accelerometer for measuring acceleration, and a three-axis magnetometer for measuring magnetic fields. The data from the IMU can be fused to obtain unbiased rotation angles that can then be applied for the purpose of coordinate system transformations.

Developing processing algorithms to detect road surface anomalies from smartphone sensor data is quite challenging. Smartphone sensor properties, car suspension systems, driving behavior, and speed affect the signal pattern when passing over any road anomaly since they are contaminated with biases and noise. Threshold-based approaches have been examined to minimize these problems, and they have been evaluated in several studies. The results of these studies were not reliable as a robust and inclusive detection approach. The machine-learning approach, which has been applied by some studies, was able to overcome some limitations encountered by threshold-based approaches. However, the proposed methods were not inclusive and did not yield a robust solution. For example, supervised approaches, such as SVM, required many trained data sets to cover all possible scenarios for classification. However, by integrating both approaches (threshold-based and machine learning-based approaches), a hybrid approach is developed which can potentially overcome the limitations of each individual approach to detect road surface anomalies from smartphone sensors.

**Figure 3:**
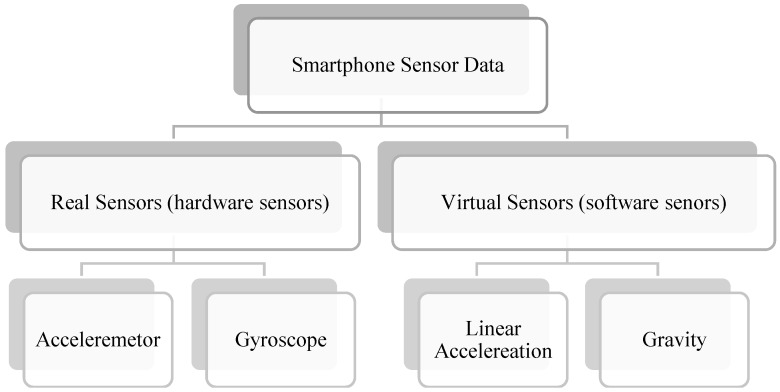
Available motion sensors on current smartphones.

Additionally, as seen in Table 1, most of the studies employed a single sensor (e.g., an accelerometer) to detect road surface anomalies. Figure 3 illustrates all available motion sensors on currently trending smartphones. Linear acceleration and gravity are the new software-based (virtual) sensors which have been recently integrated into high-end smartphone devices. To improve the system’s performance, sensor fusion techniques can be used. For example, gyroscope or gravity sensors can be combined with accelerometer sensor data to strengthen the detection of road surface anomalies. In addition, accelerometer, magnetic, and gyroscope sensor data are combined in order to derive the isolated gravity vector and to exclude it from accelerometer data. Most new and high-end smartphone devices are capable of calculating linear acceleration from their sensors. Linear acceleration is the effect of acceleration on the smartphone devices excluding the earth’s gravity. As a result, the actual acceleration of the device can be determined irrespective of the device orientation.

Most studies reviewed have implemented and verified their methods in an offline mode. However, with the continued release of powerful smartphones, few studies have both developed and verified their approaches in an online mode. Mednis et al. [41] and Wang et al. [9] implemented the proposed method on an Android OS for real-time pothole detection. However, in major studies, entire preprocessing and processing steps which have been done on computers and smartphones have only been used for sensor data collection. Due to the popularity of smartphones embedded with high-performance sensors, as well as the recent increase in the enhanced capability of smartphones, complex analysis and processing of streamed data from smartphone sensors in real time are now possible and practical [53]. For online road surface anomaly detection approaches, the feasibility of implementing an online mode for smartphones should be investigated. In addition, a proper evaluation of implemented approaches for road surface monitoring on smartphones is desirable. Smartphone resource consumption analysis, such as CPU, memory, and battery usage are topics of further interest.

Due to the complexity of the processing step, post-processing using multiple sources is able to increase both the detection accuracy and decrease the rate of false detection. Some studies investigated data crowdsourcing techniques for road surface monitoring and achieved considerable improvement of the detection accuracy rate. Unfortunately, their recommended approaches were in the very early stages of development, and they suffered from the unreliability of smartphone detection and the variable nature of road surface anomalies.

Moreover, the participatory sensing from smartphone applications presents challenges, such as flawed client-server communication due to unreliable vehicular networks, limited connectivity time, and high packet rate [54]. GPS errors also complicate data accumulation because of erratic sampling [20]. In fact, the detected location derived from the GPS sensors of smartphones have uncertainty. For instance, some existing studies proved that the cellular and/or GPS positioning can result in errors ranging from several meters to 100 m (MOK et al. [55]; Zandbergen [56]; Pun-Cheng et al. [57]). Therefore, the detected location from various smartphone users for any road surface anomaly varies due to the data uncertainty. As a result, the best approach to crowdsourcing road surface anomalies from multiple sources would be a probabilistic and spatiotemporal-based approach that would overcome both the uncertainty and variability in road surface anomalies. In addition, novel technologies of data transferring, such as the RESTful (representational state transfer) architecture and data formats such as the JSON (JavaScript object notation) data format, can be utilized to minimize the packet rate and overcome the challenges in participatory sensing using smartphones.

## 4. Conclusions and Challenges

Efforts have been made to implement various methods which detect road surface anomalies using data from smartphone sensors. However, these approaches continue to face some challenges. Regarding the performance of the associated algorithms, it is difficult to compare the accuracy and performance of various approaches due to restricted availability of the reported algorithms and data sets. For many threshold-based approaches, the manner in which the threshold was set remains unclear. In addition, methods that use supervised or unsupervised learning methods need large amounts of data to train their detection model.

Therefore, a hybrid approach, which would be able to continually detect and distinguish various road surface anomalies using real-time data streaming from smartphone sensors and other geographic data, should be developed. Sensor data values should be smoothed and reoriented to allow smartphone users more freedom, as well as to increase the accuracy of detection. The ideal approach should be self-adapting and self-learning; it would be able to reconcile itself to any platform, the dynamic behavior of different vehicles, as well as various road surface conditions.

A free, cross-platform smartphone application must be developed. This application would be fully automatic and not require any user interaction, as well as allow the driver to maintain focus on the road. In addition, the power consumption of the proposed application should be minimized, perhaps by reducing the usage of GPS sensors. Specific techniques, such as robust sensor calibration readings, should also be investigated because the amount of sensor noise and accessibility of the sensor data vary in different smartphones.

Due to the increase in the number of smartphone holders, continuous monitoring and reporting of road surface anomaly events are achievable by the public. In fact, the effectiveness of road monitoring systems could be substantially improved with effective data-crowdsourcing techniques. Smartphones generally are embedded by various wireless interfaces, such as Bluetooth, Wi-Fi, and cellular networks, making them ideal for crowdsourcing purposes and pushing notifications to road users. In fact, detected anomalies can be transferred to the central server through Wi-Fi or cellular networks for further processing and driver/authority awareness. Data integration in the server would increase system accuracy by training the machine learning algorithms and data classification operations. It would also provide further opportunities to notify drivers about the road surface anomalies ahead, and government agencies about the current road surface condition for potential maintenance and rehabilitation.

Moreover, vehicular network technologies, including V2V (vehicle to vehicle) and C-V2V (Cellular-V2V) communication technologies, are trending nowadays and can play a critical role in transportation management and specifically on driving safety [58]. By more vehicles being connected to the Internet and to each other, there is potential for enhancing the processes of (1) data collection by obtaining the most updated information regarding the road surface conditions from all connected vehicles and (2) data exchange among road users by notifying drivers approaching to road surface anomalies detected by other road users beforehand through V2V and C-V2V technologies. However, these technologies are still under investigation and are not widely deployed on vehicles due to the absence of a road infrastructure [59].

## Figures and Tables

**Table 1:** List of sensors used for road surface anomaly detection.

Sensor Name	Type	Unit	Description
Accelerometer	Physical	m/s^2^	Measures the acceleration force
Gyroscope	Physical	rad/s	Measures a device’s rate of rotation
Linear Acceleration	Virtual	m/s^2^	Measures the acceleration force, excluding the force of gravity
Magnetometer	Physical	μT (T stands for Tesla)	Measures the ambient geomagnetic field
Gravity	Virtual	m/s^2^	Measures the force of gravity
Rotation	Virtual	rad	Measures the orientation of a device
GPS	Physical	Degree	Obtain location information

**Table 2:** List of sensors used for road surface anomaly detection.

Proposed Method	Employed Sensor(s)	Data Sampling Rate (Hz)	Vehicle	Smartphone Model	Distance of Experiment	Location of Data Sampling
Mohan et al. [37]	Accelerometer	310	Toyota Qualis	Windows smartphone	622 km	Bangalore and Seattle
Yagi [21]	Accelerometer/Gyroscope	100	Toyota PRIUS	iPhone	N/A	Kashiwazaki, Japan
Mednis et al. [41]	Accelerometer	100	BMW 323 touring	Samsung i5700, Samsung Galaxy s, HTC Desire HTC HD2,	174 km	Vairoga iela, Riga, Latvia
Perttunen et al. [28]	Accelerometer	38	N/A	Nokia N95 8GB	25 km	Finland
Jain et al. [29]	Accelerometer	N/A	Bus, Auto rickshaw, cycle rickshaw, motorcycle and car (models were not mentioned)	4 different Android-based smartphones (models were not mentioned)	678 km	New Delhi, India
Bhoraskar et al. [27]	Accelerometer	50	Suzuki access 125, Auto rickshaw	Google Nexus S, HTC Wildfire S	N/A	IIT Bombay campus
Douangphachanh and Oneyama [32]	Accelerometer	100	Toyota Vigo 4WD, pick up, Toyota Camry, Toyota Vigo 2WD, Toyota Yaris	Samsung Galaxy Note 3, Galaxy S3, LG 4X HD	N/A	Vientiane, Laos
Sinharay et al. [19]	Accelerometer	4–6	N/A	Google Nexus S	N/A	Kolkata, India
Douangphachanh and Oneyama [22]	Accelerometer/Gyroscope	100	Toyota Vigo 4WD, pick up, Toyota Camry, Toyota	Samsung Galaxy Note 3, Galaxy S3, LG 4X HD	N/A	Vientiane, Laos
Seraj et al. [34]	Accelerometer	5	N/A	N/A	14 km	N/A
Vittorio et al. [38]	Accelerometer/Gyroscope	47 and 93	Five different types of cars	Samsung Galaxy S2	100.3 km	Vlora, Albania
Sebestyen et al. [33]	Accelerometer	90	N/A	N/A	N/A	N/A
Wang et al. [9]	Accelerometer	60				
Nomura and Shiraishi [42]	Accelerometer	100	N/A	N/A	N/A	N/A
Yi et al. [43]	Accelerometer	80	Toyota Camry	Sony Xperia, HTC Desire, HTC Hero	N/A	N/A
Mohamed et al. [30]	Accelerometer/Gyroscope	N/A	Volkswagen Jetta, Chevrolet Aveo	Lumia 820	N/A	N/A
Harikrishnan and Gopi [36]	Accelerometer	50	Maruti swift	N/A	N/A	India
Singh et al. [39]	Accelerometer	10	Toyota Etios	Nexus 5, Samsung S5, Samsung Note 3, Moto E, Samsung S4 mini	220 km	Chandigrah, India
Silva et al. [40]	Accelerometer	50	Three different models	Two different models	N/A	Braga-Portugal

**Table 3:** List of sensors used for road surface anomaly detection.

Proposed Method	Employed Technique(s)	Approaches	Length of Analyzing Window
Mohan et al. [37]	Threshold-based	For speed >25 km = 0.8 g and for speed <25 z-sus (sustained dip in vertical component of accelerometer data	seven samples for speed of less than 25 km/h
Yagi [21]	Threshold-based	Standard deviation of z-values with different window time	50 ms
Mednis et al. [41]	Threshold-based	Z-THERESH = 0.4 g, Z-DIFF = 0.2 g, STDEV(Z) = 0.2 g, and G-ZERO = 0.8 g	one sample
Perttunen et al. [28]	Machine learning	Support Vector Machine (SVM)	0.5 s∼2 s
Jain et al. [29]	Machine learning	Support Vector Machine (SVM)	N/A
Bhoraskar et al. [27]	Machine learning	K-means Clustering and Support Vector Machine (SVM)	N/A
Douangphachanh and Oneyama [32]	Machine learning	Linear Regression	N/A
Sinharay et al. [19]	Threshold-based	The rate change of z values in acceleration values	1 s
Douangphachanh and Oneyama [22]	Machine learning	Linear Regression	N/A
Vittorio et al. [38]	Threshold-based	Comparing the difference of maximum value and minimum value of vertical acceleration impulse in the defined unit of time with an adaptive threshold	5 samples
Seraj et al. [34]	Machine learning	Features extracted in time domain, frequency domain and wavelet decomposition and SVM used for feature classification	256 samples and 170 samples
Sebestyen et al. [33]	Threshold-based	Adaptive threshold based on the lowest, highest and average values of accelerometer data in predefined window length	one sample
Wang et al. [9]	Threshold-based	Approach proposed by Mednis et al. [41] with adaptive threshold	one sample
Nomura and Shiraishi [42]	Threshold-based	0 < roughness index (RI) < 1 for σ = 0.0190 m/s^2^ and 0 < RI < 2 for σ = 0.0428 m/s^2^	one sample
Yi et al. [43]	Threshold-based	Two steps of pothole verification based on the standard deviation of sensor data $\sigma_{\left(i-1\right)}$ < 2 × $\sigma_{i}$ and $\sigma_{\left(i-1\right)}$ < 2.5 × $\sigma_{event}$	0.5 s
Mohamed et al. [30]	Machine learning	SVM with three different kernel functions (RBF, MLP, and polynomial).	N/A
Harikrishnan and Gopi [36]	Threshold-based	Fitting Gaussian models to the normal roads and comparing the accelerometer sensor data value in the z direction with the mean of fitted model.	N/A
Singh et al. [39]	DWT	Measuring signal pattern similarity	N/A
Silva et al. [40]	Machine learning	Using different supervised classification approaches (gradient boosting (GB), decision tree (DT), multilayer perceptron classifier (MPL), Gaussian Naive Bayes, and linear SVC) and comparing the detection accuracy	125 samples

**Table 4:** Performance evaluation of reviewed studies investigating road surface anomalies from smartphone sensors.

Proposed Method	Performance Evaluation
Mohan et al. [37]	For a speed of less than 25 km/h, the rate of the false negatives is 29% (well-oriented sensor) and 37% (virtually oriented). However, for a speed of more than 25 km/h, the rate of false negatives is 41% (well-oriented sensor) and 51% (virtually oriented).
Yagi [21]	Not provided.
Mednis et al. [41]	The accuracy of the overall system is approximately 90%. However, the outcome of Z-DIFF and STDEV-Z approaches are highly dependent on the frequency and timing of data.
Perttunen et al. [28]	The confusion matrix for the best result indicates that this approach has approximately 80% accuracy.
Jain et al. [29]	The results indicate approximately 75% accuracy.
Bhoraskar et al. [27]	For bump detection, the algorithm gets zero false positives and 10% false negatives.
Douangphachanh and Oneyama [32]	The R^2^ values in their estimation were between 0.721 and 0.869 for different cars when the smartphones were located in the box near gearshift.
Sinharay et al. [19]	The accuracy of the system is 80% with 20% false positives.
Douangphachanh and Oneyama [22]	The R^2^ values in their estimation indicated significant improvement compared to the previous study.
Vittorio et al. [38]	Right positive rate was higher than 80% whereas the false positive rate was lower than 15%.
Seraj et al. [34]	90% accuracy ratio for detecting severe anomalies regardless of vehicle type and road location.
Sebestyen et al. [33]	The accuracy of the anomaly detection algorithm implemented in this study is about 80%.
Wang et al. [9]	In experiments, the results indicate the accuracy of the proposed approach is 100% without false positives.
Nomura and Shiraishi [42]	94% accuracy rate for classifying road segments into different roughness levels (detection rate for road surface anomaly detection was not provided).
Yi et al. [43]	Numerically, compared with z-component, the RMSEs (root mean square deviation) are 0.01 m/s^2^ of the batch mode and 0.03 m/s^2^ of online mode.
Mohamed et al. [30]	75.76% by applying RBF kernel function, 66.67% by applying MLP kernel function, and 87.88% by applying polynomial kernel function.
Harikrishnan and Gopi [36]	The estimation error is 34.8% for the speed of 15 km/h and 1.6% for the speed of 20 km/h. The estimation error increases as the speed goes above 20 km/h.
Singh et al. [39]	88.66% detection rate for potholes and 88.89% detection rate for bumps.
Silva et al. [40]	Scores of GB = 0.8705, DT = 0.8071, MLP = 0.7868, GNB = 0.7385, and linear SVC = 0.4619.

**Table 5:** Smartphone placement dependency considerations for each approach.

Proposed Method	Considering Smartphone Mounting Dependency
Mohan et al. [37]	Back and middle seats, dashboard, and hand-rest of vehicle
Yagi [21]	Front dashboard
Mednis et al. [41]	Front dashboard
Perttunen et al. [28]	Windshield rack
Jain et al. [29]	Pants pocket, front dashboard, near the gearbox, near the rear car speakers
Bhoraskar et al. [27]	Not defined
Douangphachanh and Oneyama [32]	Front dashboard, near the gearshift, inside driver’s pocket
Sinharay et al. [19]	Front dashboard
Douangphachanh and Oneyama [22]	On the dashboard, in the box near the gearshift, and inside driver’s pocket
Vittorio et al. [38]	Front dashboard
Seraj et al. [34]	Fixed on the windshield
Sebestyen et al. [33]	Front dashboard
Wang et al. [9]	Not defined
Nomura and Shiraishi [42]	Front dashboard
Yi et al. [43]	Front dashboard and windshield rack
Mohamed et al. [30]	Not defined
Harikrishnan and Gopi [36]	Not defined
Singh et al. [39]	Not defined
Silva et al. [40]	Not defined
